# Idiopathic Pulmonary Fibrosis: A Case of Mistaken Identity

**DOI:** 10.7759/cureus.6164

**Published:** 2019-11-15

**Authors:** Jeffrey A Miskoff, Moiuz Chaudhri

**Affiliations:** 1 Internal Medicine, Jersey Shore University Medical Center, Neptune City, USA; 2 Internal Medicine, Shore Pulmonary, New Jersey, USA

**Keywords:** diffuse parenchymal lung diseases, interstitial lung diseases, usual interstitial pneumonia, idiopathic pulmonary fibrosis

## Abstract

Diffuse parenchymal lung diseases (DPLD), also known as interstitial lung diseases (ILD), are a group of lung disorders affecting alveolar epithelium, pulmonary capillary endothelium, and surrounding lung tissue. Over time due to injury, the tissue around the air sacs becomes fibrotic leading to poor oxygen exchange, eventually resulting in the patient experiencing shortness of breath. This case describes a 69-year old male who presented in 2017 with a complex clinical picture involving both cardiac and pulmonary systems. Drug toxicity was initially thought to be the cause of the patients interstitial lung process; however, ultimately, a diagnosis of UIP was made.

## Introduction

Diffuse parenchymal lung diseases (DPLD) are a group of conditions that affect lung parenchyma; there are more than 140 different infectious and non-infectious conditions with the capability of affecting endothelium, interstitium, and epithelium of the lung. Over time, these conditions can increase cellularity and the amount of connective tissue present in the lung. Usual interstitial pneumonia (UIP) is a specific form of DPLD presenting with fibrotic and inflammatory changes in the lung. UIP is a common pathological finding seen in patients with a working diagnosis of idiopathic pulmonary fibrosis (IPF). According to the American Thoracic Society (ATS), IPF is a specific form of chronic fibrotic interstitial pneumonia which is limited to the lung and presents with the histologic appearance of UIP based on lung biopsy [1,2]. Therefore, the purpose of this case was to highlight the diagnostic challenges associated with IPF due to similarities between the different ILDs. The patient was ultimately diagnosed with UIP, which carries the worst prognosis with a mean life expectancy of 3.8 years [1]. 

## Case presentation

**Figure 1:**
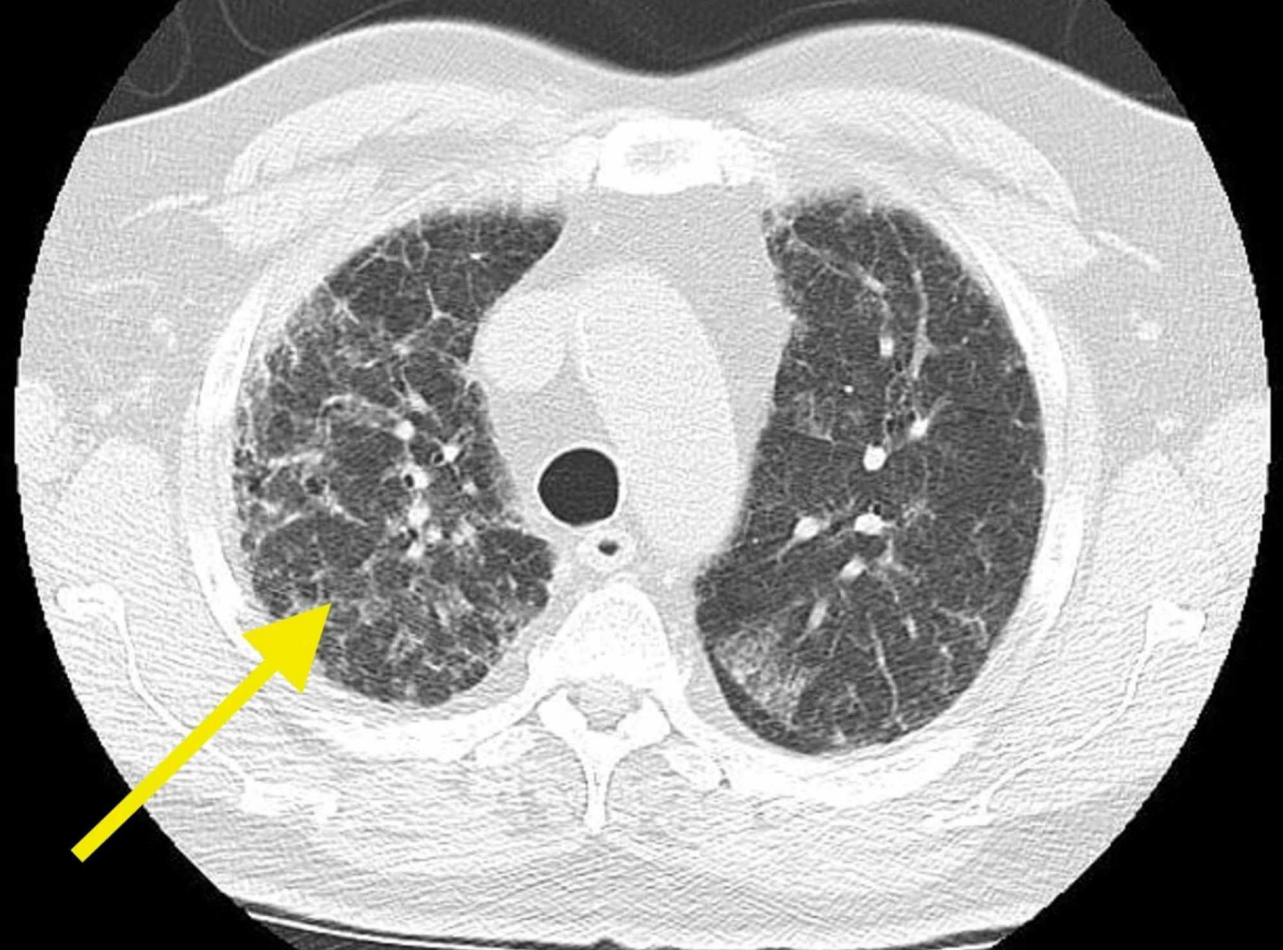
Possible amiodarone lung consolidation toward the periphery (arrow).

**Figure 2:**
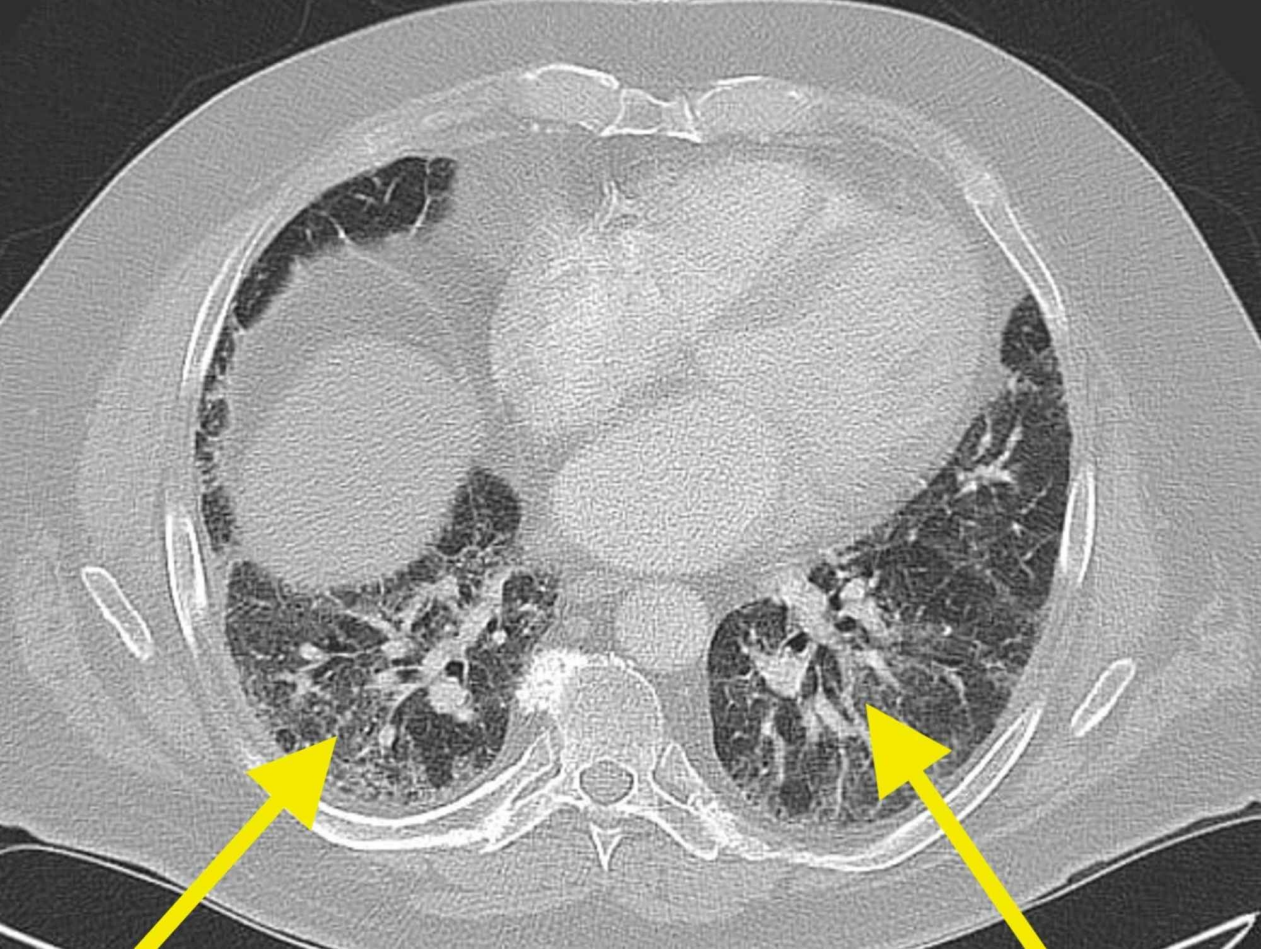
Diffuse mosaic pattern with evolving interstitial lung changes.

**Figure 3:**
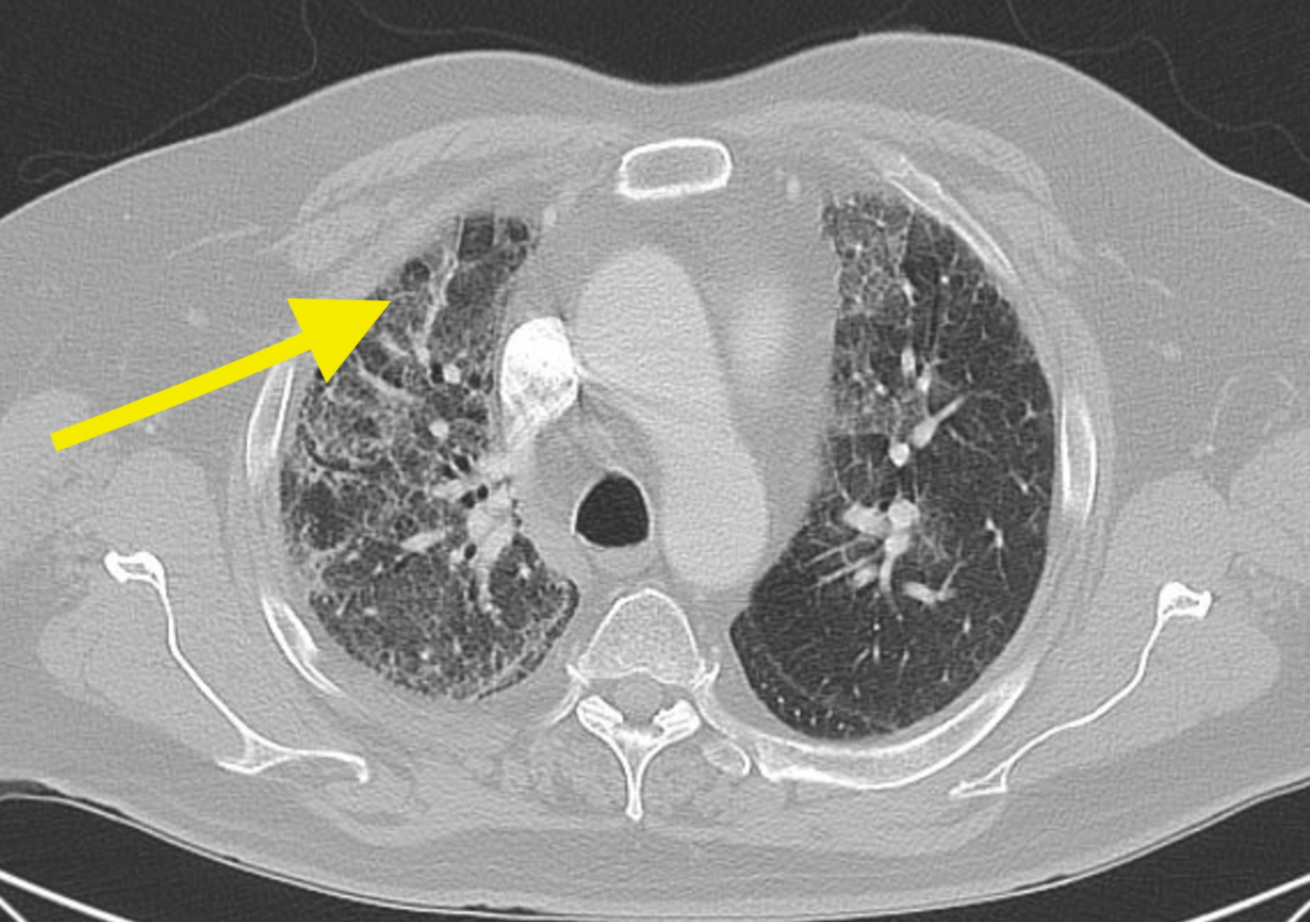
Bandlike cystic changes similar to irreversible honeycombing around the right lung.

A 69-year-old male presented to our care in February 2017 for an abnormal chest x-ray, dry cough, and worsening shortness of breath (Figure 1). Past medical history included myocardial infarction, paroxysmal atrial fibrillation, hypertension, coronary artery disease, mitral valve regurgitation, subdural hematoma, and diabetes mellitus. The patient stopped tobacco at the age of 33. An abnormal chest x-ray depicted interstitial changes attributed to probable amiodarone toxicity per the patients' cardiologist. The patient had tried several asthma medications without a proven history of obstructive lung or airway disease and without clinical benefit. Subsequently, the patient was scheduled for a chest computed tomography (CT) without contrast, which showed a diffuse mosaic pattern with evolving interstitial lung changes (Figure 2). Also, bandlike cystic changes similar to irreversible honeycombing around the right lung were noted (Figure 3). Two weeks later, the patient underwent a repeat chest x-ray, which depicted worsening bilateral interstitial markings predominantly at the bases. During the six-minute walk test (6MWT), the patient's lowest peripheral capillary oxygen saturation (SpO2) was 88% at the 2-minute mark. The oxygen saturation increased to 97% with 2 liters of Oxygen gas per minute inhalation. Lastly, he tested normal (PiMM) for alpha-1 antitrypsin deficiency (AATD) along with fractional exhaled nitric oxide (FeNO) of 5 parts per billion (ppb), a normal value.

**Figure 4:**
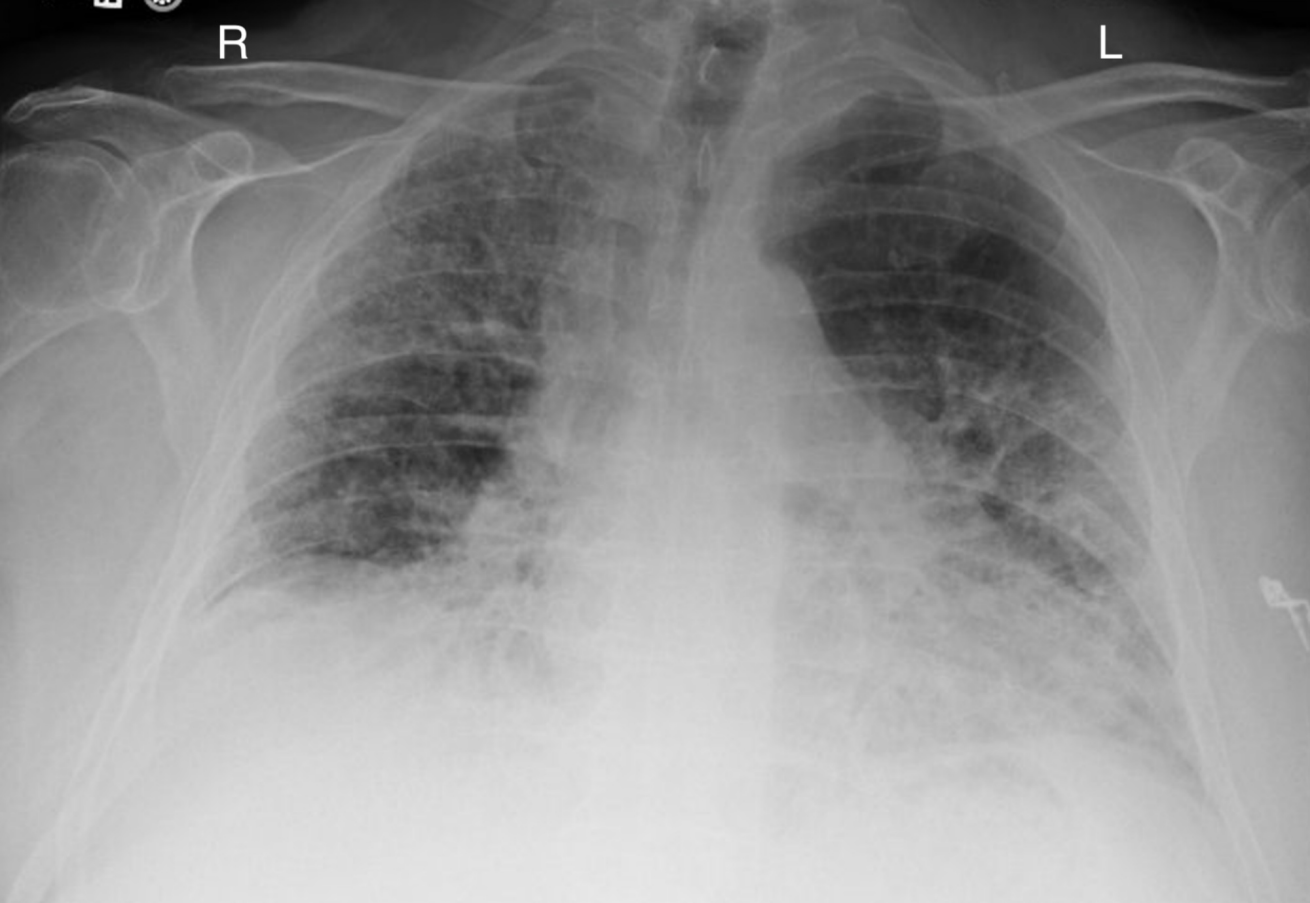
Interstitial alveolar infiltrates in the right upper and lower and left lower lobe.

Over the next few months, the patient was seen by various specialists, along with our practice, to better define his underlying condition(s). The patient was seen several times for persistent hemoptysis, along with worsening dyspnea. At that time, the patient was taking rivaroxaban and sotalol; however, secondary to hemoptysis, apixaban was subsequently tried but ultimately was held because of recurrent hemoptysis. In early 2019, the patient was sent for a chest CT, which illustrated asymmetrically patchy, bilateral ground-glass opacities raising the suspicion of possible pulmonary fibrosis, pneumonia, pneumonitis, or asymmetric pulmonary edema. Despite being managed medically, the patient continued to report shortness of breath and cough, which became productive over time. In March 2019, a chest x-ray revealed interstitial alveolar infiltrates in the right upper and lower and left lower lobe (Figure 4), which were not present on a previous chest x-ray, October 2018.

In April 2019, the patient underwent bronchoscopy, and bronchial washings from the right lung were sent for analysis. The sample was positive for pigmented macrophages, mixed inflammatory cells and squamous cells, and negative for malignant cells. The patient underwent a second bronchoscopy on May 8th, 2019, which did not show any malignancy. However, transbronchial biopsies were consistent with UIP, and the Envisia Genomic Classifier was utilized to support these findings. 

## Discussion

Interstitial lung disease (ILD) is a group of fibrotic lung disorders with an estimated prevalence of 67-81 per 100,000 people [2,3]. ILDs can be categorized into four groups: 1) ILD of known causes (e.g., connective tissue diseases, environmental or occupational exposures along with drug-induced condition; 2) sarcoidosis; 3) idiopathic interstitial pneumonia; 4) lymphangioleiomyomatosis, Langerhans' cell histiocytosis, alveolar lipoproteinosis or eosinophilic pneumonia [3,4]. Idiopathic pulmonary fibrosis (IPF) is a specific form of chronic progressive fibrosis of the lung tissue and defined by the histopathological and or radiographic pattern of UIP [5,6]. Specifically, IPF should be considered in patients presenting with progressive shortness of breath, cough, and bibasilar inspiratory crackles without a known cause [5,6].

Our patient presented with similar findings as outlined by the American Thoracic Society (ATS). One of the critical findings of ILD is the usual interstitial pneumonia (UIP) pattern seen in the specimen collected via the surgical lung biopsy or by high resolution computed tomography of the chest (HRCT) [4,3]. Envisia Genomic Classifier is a noninvasive diagnostic test that utilizes an RNA sequencing assay to amplify exonic transcriptions obtained from 15 ng of RNA from 3-5 transbronchial lung biopsies (TBB) [3]. 

In a recent clinical validation study [Bronchial Sample Collection for a Novel Genomic Test (BRAVE)] 29 sites assessed the Envisia Genomic Classifier’s ability to identify the UIP pattern required for IPF diagnosis. Genomic Classifier achieved a specificity of 88% (95% CI 70-98) and sensitivity of 70% (95% CI 47-87) [7]. Furthermore, the study exhibited 42 patients with inconsistent UIP pattern on HRCT; these individuals had 81% positive predictive value (95% CI 54-96) when evaluated with the Genomic Classifier [7]. In contrast, high resolution computed tomography (HRCT) imaging has a sensitivity of 43% for diagnosing UIP. In a clinical utility analysis [Clinical Utility Analysis of a UIP Genomic Classifier in the BRAVE Trial (CATALYST)], authors reported finding 86% agreement (95% CI 78-92) between diagnosis reached using the classifier and the histopathologic data [8]. Lastly, the classifier improved diagnostic confidence in 48 patients who had non-diagnostic pathology or not classifiable fibrosis (63% vs. 42%, p=0.0412) [8].

The patient presented to our care in 2017 after cardiology requested an evaluation for probable amiodarone toxicity. After amiodarone was held and prednisone 20mg daily started, the patient improved clinically. Initially, the patient had a pulmonary function test (PFT) associated with a restrictive pattern and diffusion abnormalities that corrected for alveolar ventilation. Subsequent spirometry revealed moderate restriction with Forced Expiratory Volume in 1 second (FEV-1) and Forced Vital Capacity (FVC) of 72% and 66%, respectively. However, the last office spirometry revealed his flow rates worsened with FEV-1 and FVC of 63% and 56%, respectively. 

Prior to being diagnosed with UIP, clinically and radiographically, it was felt that the patient had Nonspecific Interstitial pneumonia (NSIP) or perhaps Chronic Hypersensitivity pneumonitis (CHP). Uncertainty in diagnosis arises from significant overlap in the clinical presentation, along with similarities seen on radiographic imaging [1]. In 2014, two antifibrotic medications, Nintedanib and Pirfenidone, were approved by the Food and Drug Administration (FDA) for IPF/UIP [1]. Therefore, the availability of a genomic classifier is a helpful addition to aid in the accurate diagnosis of IPF, which may lead to earlier treatment and better outcomes, including slowing the reduction in FVC and potential symptoms. 

## Conclusions

Idiopathic pulmonary fibrosis is a specific form of interstitial lung disease, which leads to progressive fibrosis of lung parenchyma and eventually resulting in an irreversible decline in lung function. UIP is a common pathological finding associated with IPF, and it carries the worst prognosis with a mean life expectancy of 3.8 years. It is crucial to identify UIP early in order to initiate the most appropriate FDA approved agent(s) as early as possible. Unfortunately, the patient’s condition rapidly worsened after the official diagnosis. He passed away within 72 hours, which was 2.5 years post original presentation.
